# Development of the horizontal optocollic reflex in juvenile barn owls (*Tyto furcata pratincola*)

**DOI:** 10.1007/s00359-022-01555-0

**Published:** 2022-06-13

**Authors:** Hermann Wagner, Ina Pappe, Sandra Brill, Hans-Ortwin Nalbach

**Affiliations:** 1 Present Address: RWTH Aachen University, Institut für Biologie II, Worringerweg 3, D-52074 Aachen, Germany; 2 Present Address: Universitätsklinik Für Anaesthesiologie, Waldhörnlestrasse 22, D-72072 Tübingen, Germany; 3 Max-Planck-Institut für Biologische Kybernetik, Max-Planck-Ring 11, D-72076 Tübingen, Germany

**Keywords:** Nystagmus, Optokinetic, Optomotor, Saccade, Binocular

## Abstract

Adult barn owls and primates possess an almost symmetric monocular rotational horizontal optocollic reflex. In primates, the reflex is initially asymmetric and becomes symmetric with time after birth. The condition in barn owls has not been studied so far. Here, we present data on the development of this reflex in this bird. We tested juvenile barn owls from the time before they open their eyes after hatching to the time they reach adult feather length. Wide-field visual patterns served as stimuli. They were presented at different rotational speeds in binocular and monocular settings. The binocular horizontal optocollic responses of juvenile barn owls were symmetric and adult-like on the first day that the birds responded to the stimulus. The monocular responses showed different rates of development in respect to stimulus velocity and stimulus direction. For velocities up to 20 deg/s, the monocular reflex was also adult-like on the first day that the birds responded to the stimulus. An initially higher asymmetry for 30 deg/s compared to adults disappeared within about two weeks. The development at even higher velocities remained unclear.

**Supplementary Information:**

The online version contains supplementary material available at 10.1007/s00359-022-01555-0.

## Introduction

Practically, all visual animals follow wide-field visual stimuli with their eyes, head and body (for reviews see, e.g., Huang and Neuhaus 2008; Masseck and Hoffmann 2009; Knapp et al. 2013; Carde 2021). This following behavior is called optomotor reflex (Carpenter 1988; Gioanni 1988). The reflex may be specified as optokinetic (OKR, eye movement based), optocollic (OCR, head movement based) or optomotor (OMR, based on movements of the whole body). Barn owls can rotate their eyes only by a few degrees (Steinbach and Money 1973; Du Lac and Knudsen 1990; Nieder and Wagner 2000; Iwaniuk et al. 2008; Netser et al. 2010), and show a pronounced optocollic response when stationary. Note that when we mention the OCR in this work, we always mean the horizontal rotational OCR (hOCR); if we mention “owl”, we always mean “barn owl”.

Primates possess large eye movements; their response to wide-field visual stimuli is predominantly an OKR (Masseck and Hoffmann 2009). The reflex is characterized by a slow-phase segment during which the subject follows the movement of the wide-field stimulus, and fast return saccades. The resulting sawtooth-like pattern of gaze is called nystagmus. It was recognized early on that the nystagmus is an innate behavior. It is not fully developed at birth, matures during early postnatal life, and may be influenced in its development by environmental factors (Simon 1954; Schor 1993). While the response to binocular stimulation is typically stable and of high gain, the situation for monocular stimulation differs. Primates exhibit a symmetric horizontal OKR under monocular stimulation. In other words, the reaction upon stimulation in the nasal-to-temporal direction (N–T) is as high as the reaction upon stimulation in the temporal-to-nasal (T–N) direction (e.g., van den Berg and Collewijn 1988; Distler et al. 1999). By contrast, birds with laterally placed eyes typically show an asymmetric hOCR with a higher T–N than N–T gain (e.g., Mowrer 1936; Gioanni et al. 1981; Wallman and Velez 1985). The reaction of adult barn owls (*Tyto furcata pratincola*) is somewhere in between, but closer to that of primates than to that of chickens (Wallman and Velez 1985; Distler et al. 1999; Wagner et al. 2021). Adult owls have a symmetric hOCR for low stimulus velocities (< 20 deg/s). The response becomes moderately asymmetric for velocities between 20 and 40 deg/s.

In primates and cats, the symmetry of the reflex develops gradually after birth or eye opening (Schor 1993; Distler and Hoffmann 2003). The reflex is initially asymmetric and becomes symmetric with time. The duration of development is shorter for low stimulus velocities. It lasts from three to four weeks in macaques to more than two years in humans (Naegele and Held 1982; Lewis et al. 2000; Distler and Hoffmann 2003). Symmetry is observed after cortical inputs make synapses in the sub-cortical network underlying the reflex (Distler and Hoffmann 2003). It is unclear whether a similar development exists in owls.

**Fig. 1:**
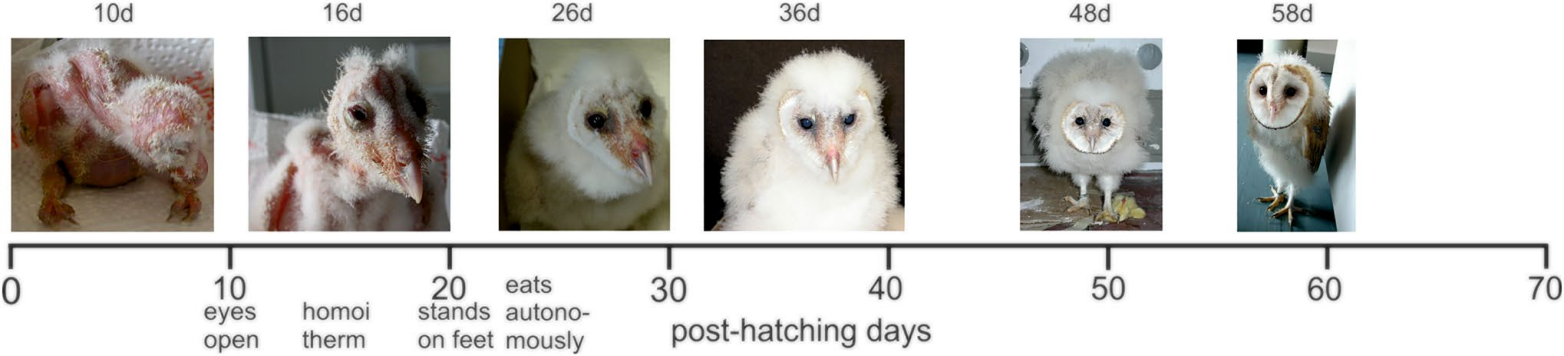
Post-natal development of the barn owl. Six stages of development are shown from left to right. The age and major developmental steps are mentioned. *d* = post-hatching day

Barn owls are altricial. They are born blind, open their eyes between post-hatching days (P or PHD) 10 and 12, grow fast, can stand on their feet around P20, and start to fly around P60 (Bunn et al. 1982; Koeppl et al. 2005; Krings et al. 2018; Roulin 2020) (Fig. 1). Feather length reaches adult values at P67 (Shawyer 1998). We tested juvenile owls from P9 to P65 to study the development of their OCR. Analyses showed that the OCR in juvenile owls is adult-like and symmetric for low stimulus velocities just after eye opening. The OCR is initially more asymmetric than in adults for high stimulus velocities, and becomes adult-like within a short time after eye opening.

## Materials and methods

Six tame, hand-raised barn owls participated in the experiments. The birds (codes: F, G, H, I, J, K) were taken out of the nest shortly before or shortly after the time when the eyes open, and raised by hand. In this way, the owls became tame and worked readily with the experimenters. The birds required thermal support until they were about 15 days old (Fig. 1). Before this age, young owls sit on their metatarsi and are unable to walk. Around P20, the owls become able to stand upright and walk (Fig. 1). The owls were calm before they could walk, then became increasingly agile. The time between P20 and P30 is critical (Wagner, unpublished information). Untamed birds start to show aggressive behavior towards strange subjects from this time on. Thus, it is important to keep close human contact to the juveniles from about P20. Birds of this age wander around and hide. However, if they are frequently handled by people, they may become very tame. The agility made it more and more difficult to record OCRs after about P30, because the cooperation of the owls during the experiments became variable.

### Set-up and stimuli

**Fig. 2:**
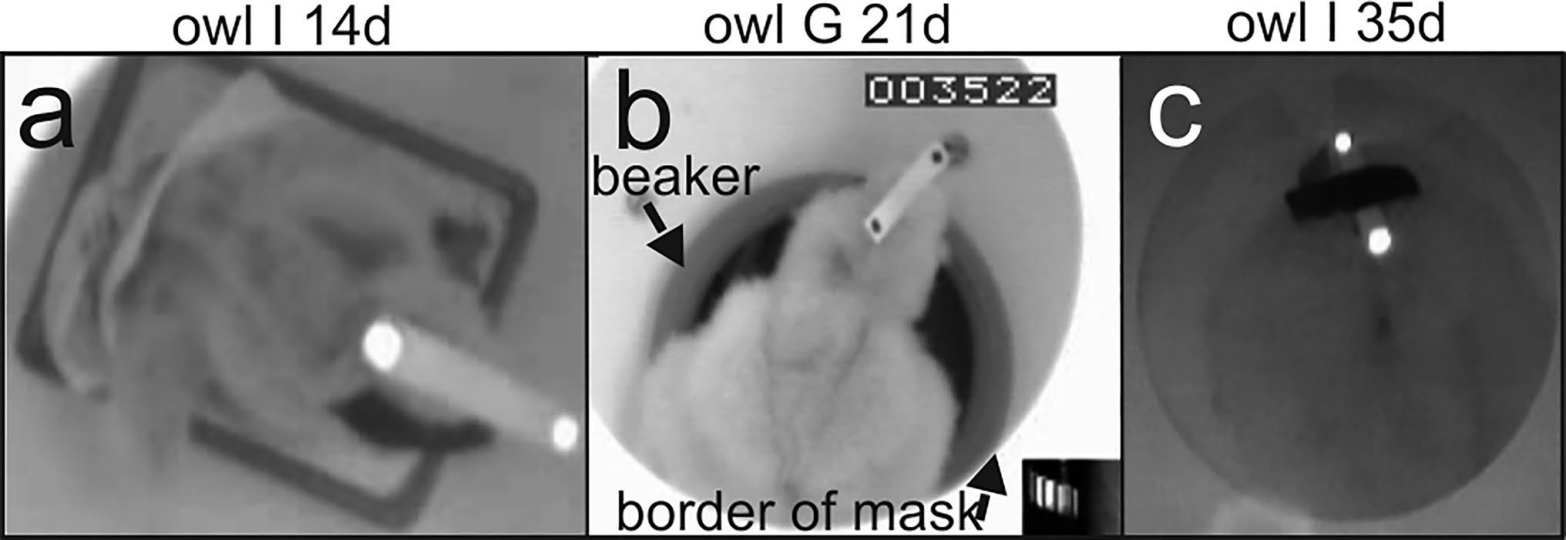
Juvenile barn owls in the set-up. **a** Young bird in a staining dish with white stripe carrying reflecting spots and the right eye covered with black adhesive tape. **b** P21 bird sitting in a beaker (arrow) with white stripe with black dots on the top of the head. Frame number, gearwheel and the border of the mask (arrow) are also shown. **c** Barely visible older bird with bright reflecting spots mounted on a stripe of cardboard that was fixed to the head with black adhesive tape. The bright **a**, **c** or dark **b** spots were used for reconstruction of head azimuth

The set-up and the stimuli were the same as in the work with the adult barn owls (Wagner et al. 2021). Briefly, visually induced OCRs were elicited with a rotating drum (diameter 64 cm, height 46 cm, angle subtended in elevation 70 degrees as seen from half height). The drum carried the stimulus pattern. The stimulus pattern consisted either of evenly horizontally and vertically spaced squares (2.7 × 2.7 degrees as seen from the center of the drum) (Nalbach 1992) or of a white-and-black striped pattern (horizontal wavelength 10 degrees as seen from the center of the drum). The high-contrast pattern was diffusely illuminated from outside and had an average light intensity of 27.3 cd/m^2^. The animal was positioned in the middle of the drum during an experiment. Young babies up to the age of about 20 days were typically placed in a staining dish (Fig. 2a). Older birds were typically placed in a beaker with its size fitted to the size of the animal (Fig. 2b (see arrow), Fig. 2c). Other containers were also tested. All shared the property that they helped to stabilize the posture of the juveniles. The animals could move the body and the head in each of the containers tested. Sheets of paper at the bottom and top of the drum masked stationary contours. In this way, the reaction of the animals corresponded to a “stare” or “delayed” OCR (for details see Türke et al. 1996). A 16.5 cm wide circular hole in the center of the top of the rotating drum (see brighter circles marked by the arrow in Fig. 2b) allowed to videotape the movements of the owl’s head.

### Data recording

Recording of monocular and binocular OCRs took place between May 1992 and June 1993. A recording session typically lasted one hour. For recording monocular OCRs, either the right or the left eye of a bird was occluded. Different eye covers were used with the aim to adjust the cover optimally to the age of the bird. For example, in the photo shown in Fig. 2a, the right eye of the bird is covered with black adhesive tape. All eye covers worked similarly well. In older juveniles (*p* > 50), the eye cover was fastened to a holder. The holder had been fixed to the animal’s skull under anesthesia with dental cement [for further details on surgery and anesthesia see Wagner (1993)]. The surgery and the experiments were carried out under a permit issued by the Regierungspräsidium Tübingen, Germany. Recording gear was mounted shortly before an experiment and removed immediately afterwards.

Reactions were recorded without earlier training. Data for a broad variety of conditions were collected: different stimulus types (binocular, monocular N–T, monocular T–N), different ages (P11–P65), and different drum velocities (5, 10, 15, 20, 30, 40, 60, 80 deg/s) (for details see “Results”). If more than one stimulus velocity was tested on a given recording day, stimuli were presented in a pseudo-random order. Stimuli moving in clockwise and counter-clockwise direction were applied in alternation.

A potentiometer attached to the shaft of the drum served to determine stimulus position. A stripe of cardboard with two reflection spots at its ends helped to measure head rotations. The stripe was temporarily taped to the feathers on top of the head of the owl (Fig. 2). The stripe was not moving relative to the head as assured by visual inspection. Videotaping took place from above (Fig. 2). In most cases an infrared light source illuminated the reflection spots. In other cases, the spots were painted with white color onto a black stripe or black dots onto a white stripe. The high contrast of the spots was needed for the automatic reconstruction of head position (see next section and Wagner et al. 2021).

### Data analysis

As mentioned above, we measured the development of the OCR relative to hatching by using the PHD or age as an independent variable. In some cases, we also use the term “first day of response”. This term refers to the first age at which data were recorded and the owls responded to the visual stimulus. The automatic analysis of the optocollic reactions was carried out with a temporal resolution of 80 ms (for details see Wagner et al. 2021). This analysis synchronously yielded the azimuthal orientation of the owl’s head and the azimuthal position of the pattern. These two parameters were stored together with the time after onset of analysis for further processing. The horizontal angular velocity of the head was calculated from head orientation. The beginning and the end of slow-phase segments were determined by a thresholding mechanism (for details see Türke et al. 1996). The results were controlled later by visual inspection and corrected, if necessary.

We calculated the gain that characterizes the effectiveness of the hOCR during the slow-phase segment. The gain reflected the relation of the rotational head velocity of the bird to the angular velocity of the stimulus as derived from the potentiometer data. We, thus, defined the “closed-loop gain” arbitrarily (for a discussion of intricacies see Wagner et al. 2021) as:

1 $${\text{Gain }}\left( \% \right) = \frac{{\text{Angular velocity of animal}^\prime\text{s head}}}{{\text{Angular velocity of stimulus}}} \times 100.$$

One slow-phase segment yielded one data point for the analysis. Slow-phase segments needed to have a duration of a least five sequential time points to be included into the analysis.

### Data fitting

We chose to fit the temporal development of the gains with a sigmoidal function. The reason for choosing this function was that in several cases the development started at a low gain value and reached an asymptotic upper value after some time. To us it seemed that the sigmoidal function yielded a simple approach; it has only three free parameters to which a physiological meaning may be assigned. The function was defined as follows:

2 $$y\left( x \right) = \frac{a}{{1 + e^{{\frac{b - x}{c}}} }}$$

Here, *y(x)* is the gain resulting from the fit, while *x* is PHD. The upper asymptotic value *a* corresponds to the gain value finally reached. We shall use the abbreviated term “upper value” in the following if we refer to *a*. *b* represents the inflection point of the exponential in PHDs and is a proxy for the start of development. The factor *c* influences the steepness of the function and, thus, correlates with the duration of development. The aim of the fitting was to minimize the sum of the root-mean square errors (RMSE) between the data and the sigmoidal function. Note that we did not clamp the fit to zero at PHD = 0 or any other PHD, but included only actual measurements in the fitting procedure. We chose to base the fits on the medians and not on all single data values. Controls with all data showed that results changed only marginally compared with the medians (data not shown). The “90%-PHD”, i.e., the PHD at which 90% of the upper value was reached, served as further measure for the duration of development. The same holds for the “90–50-difference”, the difference in days between the 90%-PHD and the PHD of the inflection point of *y*(*x*) (50% of the upper value).

### Statistics

As observed in the adult study (Wagner et al. 2021), most of the data presented here did not show normal distributions (data not shown). Therefore, we used nonparametric statistics, specifically the Mann–Whitney *U* test to analyze the relation of unpaired samples. Some data sets were also subjected to a correlation analysis, and some to a Wilcoxon matched pairs signed rank test (online program located at https://www.statskingdom.com/175wilcoxon_signed_ranks.html). If we refer to “adult data” in the following, we mean the data as published in Wagner et al. (2021).

## Results

In total, we analyzed 5357 slow-phase segments from the responses of six birds. The data resulted from an array of conditions: the individual birds (Table 1), clockwise and counter-clockwise stimulation, binocular and monocular stimulation, stimulus velocity and age of the birds, given in PHDs.

**Table 1:** Distribution of number of cases in respect to individual birds

Owl	All	F	G	H	I	J	K
#	5357	1095	302	411	1454	1154	941

Responses to clockwise and counter-clockwise stimulation were equivalent in binocular adults (Wagner et al. 2021). Therefore, we pooled the responses in these two conditions for the further analyses. Binocular stimulation contributed 1380 data points, monocular stimulation in the T–N direction 2335 data points, and monocular stimulation in the N–T 1642 data points. With respect to age, we attempted to record data at certain PHDs for most velocities. At the remaining PHDs we only recorded data for stimulus velocities of 10, 15 and 30 deg/s. Thus, the number of cases at the different PHDs (Table 2) and the different velocities (Table 3) differ. As consequence, we have much more data for the stimulus velocities 10, 15 and 30 deg/s than for the other stimulus velocities. The earlier data appear to provide the most reliable results and may serve as critical benchmarks for interpretation. We also present the data from the other stimulus velocities below, because no other data from juvenile owls are available. Moreover, they illustrate the development more broadly. In this sense, we regard them as supplementary data that complete the picture (for more discussion see below). With respect to individual birds, we concentrated on certain velocities for certain birds (owl I: 10 deg/s; owls G + H: 15 deg/s, owls J + K: 30 deg/s). Owl F was tested with all velocities.

**Table 2:** Distribution of number of cases in respect to age

Age	11	12	13	14	15	16	17	18	19
#	14	81	65	252	354	303	297	498	348
owls	H, K	F, H, K	F, H	F, G, I, J, K	F, G, I, J, K	H, J, I, K	H, I, J	F, G, H, I, J, K	F, G, H, I, K

Age	20	21	22	23	24	25	26	27	28
#	188	319	259	119	174	459	194	212	44
Owls	G, H, I, J	F, G, I, J, K	F, I, J	H, I, K	F, I, J	F, G, H, I, J, K	F, H, I	G, I, J, K	I

Age	29	30	31	32	33	35	36	37	38	39	40	49	50	56	65
#	102	117	14	52	186	35	124	96	27	71	80	102	55	31	85
Owls	I, J	K	H	H	I, J, K	I	J, K	J	H	J	G, J	J	K	I	K

**Table 3:** Distribution of number of cases in respect to velocity

Velocity (deg/s)	5	10	15	20	30	40	60	80
#	172	2039	713	246	1586	302	233	66
Owls	F,J, K	F, I, J, K	G, H	F, J, K	F, J, K	F, J, K	F, J, K	F, J, K

In the following, we first describe general observations of the juveniles in the stimulus set-up during the recordings. We then present the temporal development of binocular responses, and finally report responses to monocular stimulation.

### General observations of juvenile barn owls during recording

Tests with three owls started before the birds showed a reaction to the stimulus, and before they presumably opened their eyes. The eye lids are closed at birth. Then, a small slit can be seen, but it is not clear whether the birds really see something. The latter can only be inferred from behavioral reactions or through invasive methods, which we did not use. Initially, we used behavioral testing with several stimuli apart from the wide-field stimulus later used for recording optocollic data. Amongst these were stimulation with a moving stick or a moving hand. During these attempts, the owls were typically sitting in the drum on different platforms. Stimulation always lasted several minutes. When the birds were not being tested, they were maintained in a comfortable environment close to the experimenters. Therefore, tests with the very young birds could be repeated several times a day.

**Fig. 3:**
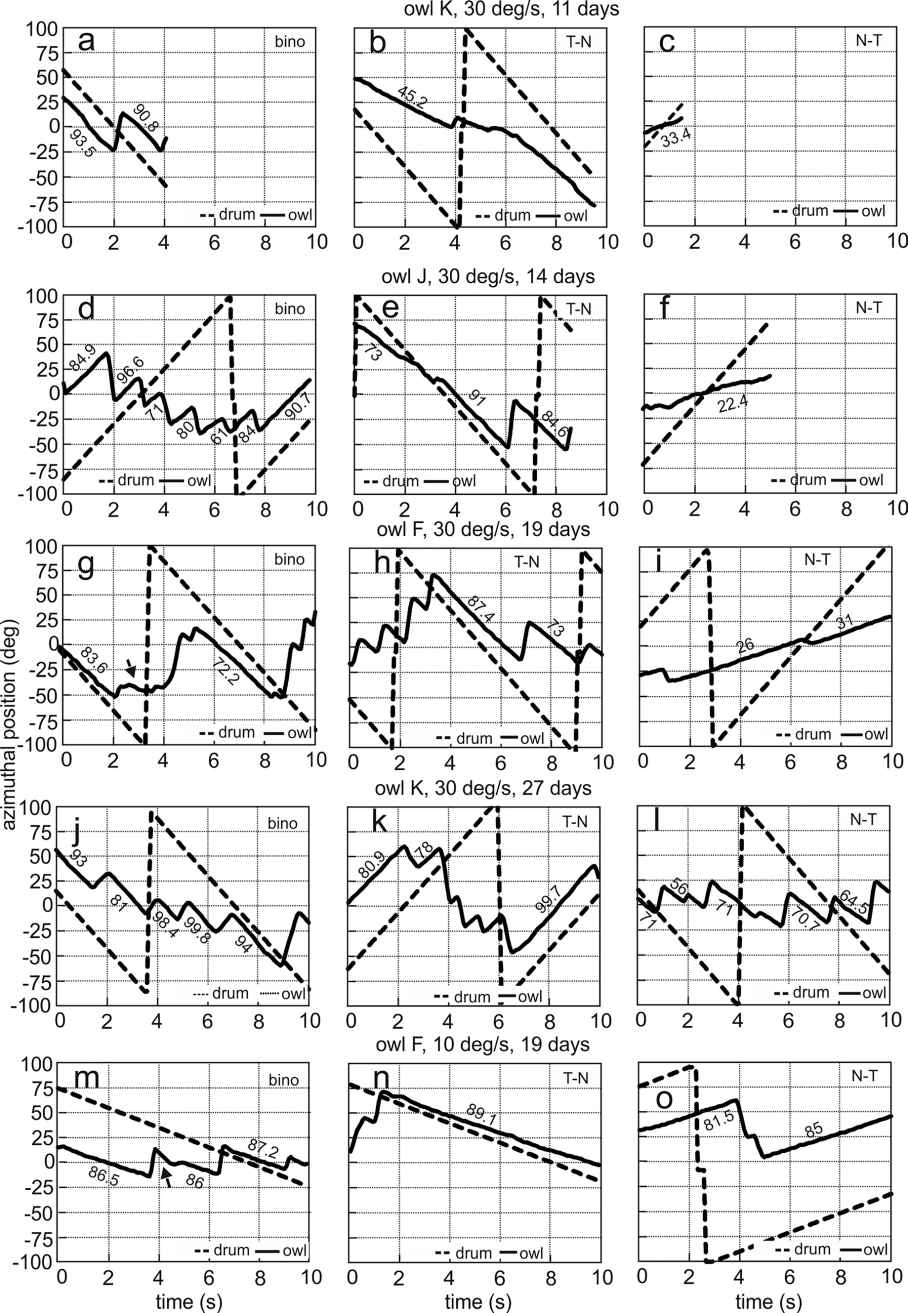
Examples of OCRs of juvenile barn owls. **a**-**o** The responses to different stimulus types at different ages and different velocities for different birds as notified in the insets or on top of the middle plots are shown. Dashed lines represent a reference position on the wide-field pattern, plotted in the range between ± 100 degrees. Note that the dashed lines from + 100 to -100 degrees and the saw-tooth-like appearance of stimulus position are due to wrapping. Solid lines signify the position of the owl’s head in azimuth. The numbers close to the individual slow-phase segments specify the gain during the respective segment. The arrows in **g** and **m** point to rare events as explained in the text

Owl K did not react to the optomotor stimulus on P9 (see video 1 in supplements) and P10. It showed the first following behaviors on P11. During the recording on P11, the bird was sitting in a beaker in the drum and was stimulated by wide-field motion (see video 2 in supplements; Fig. 3a–c). Likewise, owl F did not follow stimulus motion on P11, but did so on P12. Thus, in these two birds the very first reactions to the wide-field stimulus could be documented. Owl J was tested every day from P10 on. It first reacted to the stimulus on P13, but the first data available are from P14. In the other three birds, testing started also on P13 or P14. All six owls showed persistent reactions from P14 on (see video 3 in supplements). Interestingly, the periods during which the birds followed the stimulus were typically interrupted by periods during which the birds did not react (see video 3 in supplements). Also, apart from the rotational movements, we sometimes observed translational movements of the head (see video 3 in supplements). The latter were not further analyzed. Across owls, our data set consists of quantitative measurement from P11 to P65.

Typically, very young birds were placed in a staining dish or a beaker, and supported by soft paper for comfort, but otherwise free to move during the recording (see video 3 in supplements). It was obvious that very young birds (approximately up to P13) had difficulty in stabilizing their head. Nevertheless, the birds exhibited high-gain responses. The head was above the upper rim of the dish, with the lower jaw often touching the rim. In this situation, the head rotated and followed the rotation of the pattern. The birds could hold up their head from about P14 on (Fig. 2a). Although the birds were not yet standing on their feet, now the head did no longer touch the rim of the staining dish. The birds were calm and typically followed the stimulus. After a few more days (around P20), the birds became able to stand (Fig. 2b). At this time, the birds became more agile (Fig. 2c), and sometimes started to negotiate the staining dish. During testing, we moved the birds to a beaker adapted to the size of the birds. Note that the birds were free to move in the beaker and not restrained in any way. While the birds tolerated being seated in the beaker, their responses became more variable after P30, which typically begins a period of motor development, and exploration of the nest. Untrained birds were more easily distracted and sometimes showed no interest in the stimulus pattern (see video 4 in supplements). Nevertheless, it was possible to record data after P30 and up to P65, the last day of juvenile life covered in this work.

### Binocular optocollic responses of juvenile barn owls

**Fig. 4:**
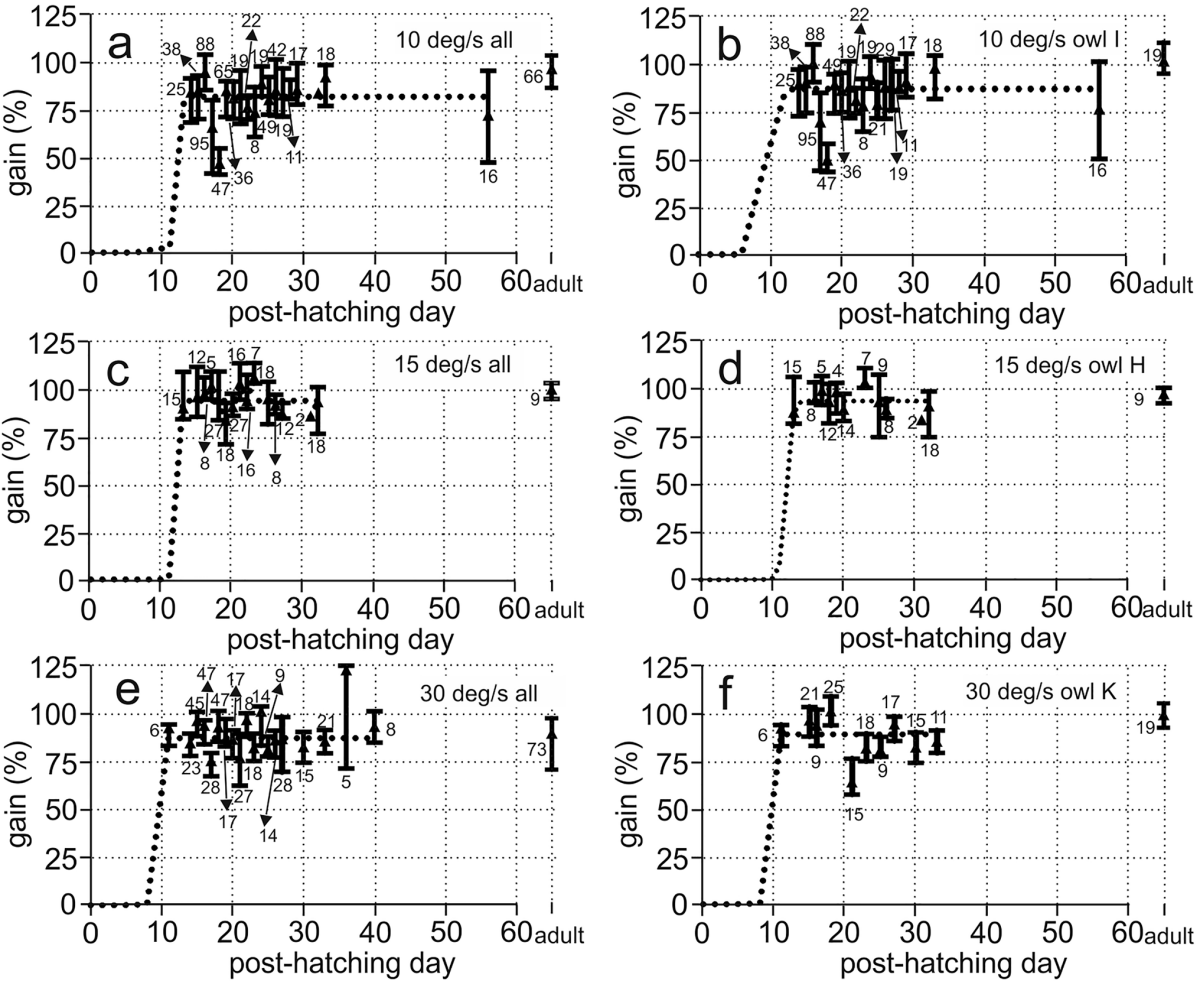
Dependence of binocular gains on age. Median data (triangles) and 1st to 3rd quartiles (lines) are shown for different days of recording (x-axis) and different stimulus velocities, including all (**a**, **c**, **e**) or only data of an individual bird (**b**, **d**, **f**). The respective fit function is shown by the dotted line. Adult data (Wagner et al. 2021) are documented for comparison in each plot on the right. The numbers specify the number of cases for each condition

**Fig. 5:**
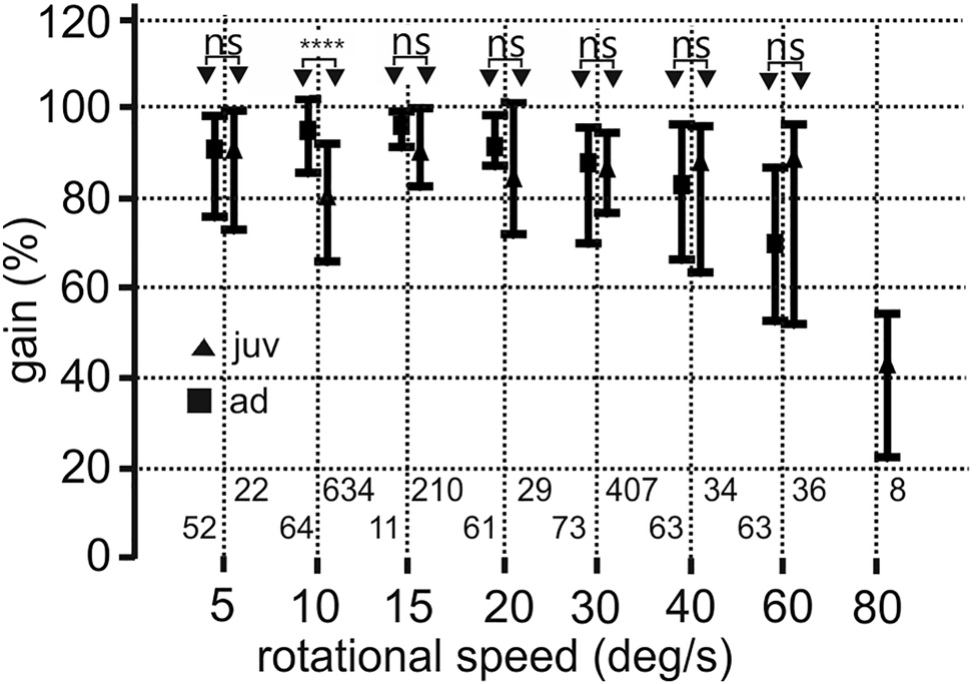
Comparison of juvenile and adult binocular OCRs. The median data together with the 1st and 3rd quartiles are shown. Note that the rotational speeds are not plotted on a linear axis. The numbers between 0 and 20% gain specify the respective numbers of cases. The data of juveniles and adults are not different (ns) for 6 out of 7 velocities and highly significantly different (****) for 10 deg/s. For 80 deg/s, only juvenile data were available

This report includes binocular data from all owls and for all stimulus velocities (Table 4). Binocular stimulation with both wide-field patterns very reliably elicited the OCR in juvenile owls of all ages. The birds showed consistent reactions to all stimulus velocities tested (Fig. 3a, d, g, j, m). Binocular gains were adult-like from the first day of response for all stimulus velocities tested (Fig. 4). In the following, we first discuss five typical examples that provide a picture of the variability of the responses (Fig. 3a, d, g, j, m). Afterwards, we present a quantitative analysis (Figs. 4, 5).

**Table 4:** Distribution of the number of cases on different conditions (binocular, N–T, T–N)

	Binocular	Owls tested	N–T	Owls tested	T–N	Owls tested
#	1380	6	1642	6	2335	6

The typical reaction of an owl to visual wide-field stimulation was to follow the stimulus by head rotation. Stimulus movement in the counter-clockwise direction elicited a counter-clockwise head rotation during the slow-following phase (Fig. 3d). Opposite (clockwise) head turning occurred with opposite (clockwise) stimulus movement (Fig. 3a, g, j, m). A slow-phase segment ended with a saccadic turn in the opposite direction to the slow-phase movement. While the owl followed the stimulus, the angular velocity of the head was almost constant. This may be concluded from the almost linear change of head azimuth with time (Fig. 3a, d, g, j, m). Gains were often 80% or higher. Only one out of nineteen slow-phase segments shown for binocular stimulation in Fig. 3 had a gain below 70% (see numbers close to the single slow-phase segments in Fig. 3a, d, g, j, m and Fig. 4).

Before analyzing the typical behavior of the birds presented so far, we point to some rare behavior. For example, a special situation is shown in Fig. 3g. Here, the first following movement had a high gain. A low-amplitude saccade followed. Then, the owl ceased to follow the stimulus for about 3 secs, before it started the next following movement (see arrow in Fig. 3g). The period during which the owl was not following the stimulus was not included in the analysis. This may be seen from the gain values noted in Fig. 3g (83.6 and 72.2). Another peculiarity occurred in the sequence shown in Fig. 3m. Here, a return saccade started at 3.68 s. After this saccade, the head movement was initially much faster than the stimulus movement for more than half a second (3.92–4.64 s, see arrow in Fig. 3m). Then a movement in the opposite direction occurred with a low velocity (4.72 to 4.96). Finally, the bird started to follow the stimulus with a gain of 86% at 5.12 s. Both, the fast head rotation from 3.92 to 4.64 s, and the movement in the opposite direction were not included in the analysis. In the other 3 examples (Fig. 3a, d, j), the owl followed the stimulus during the total time sequence. This was the typical behavior that occurred in the vast majority of cases. Note, however, that the amplitudes of the following movements varied considerably. We did not further analyze amplitudes and durations of the slow-phase segments. Instead, in this study, we concentrated on the development of gain.

**Fig. 6:**
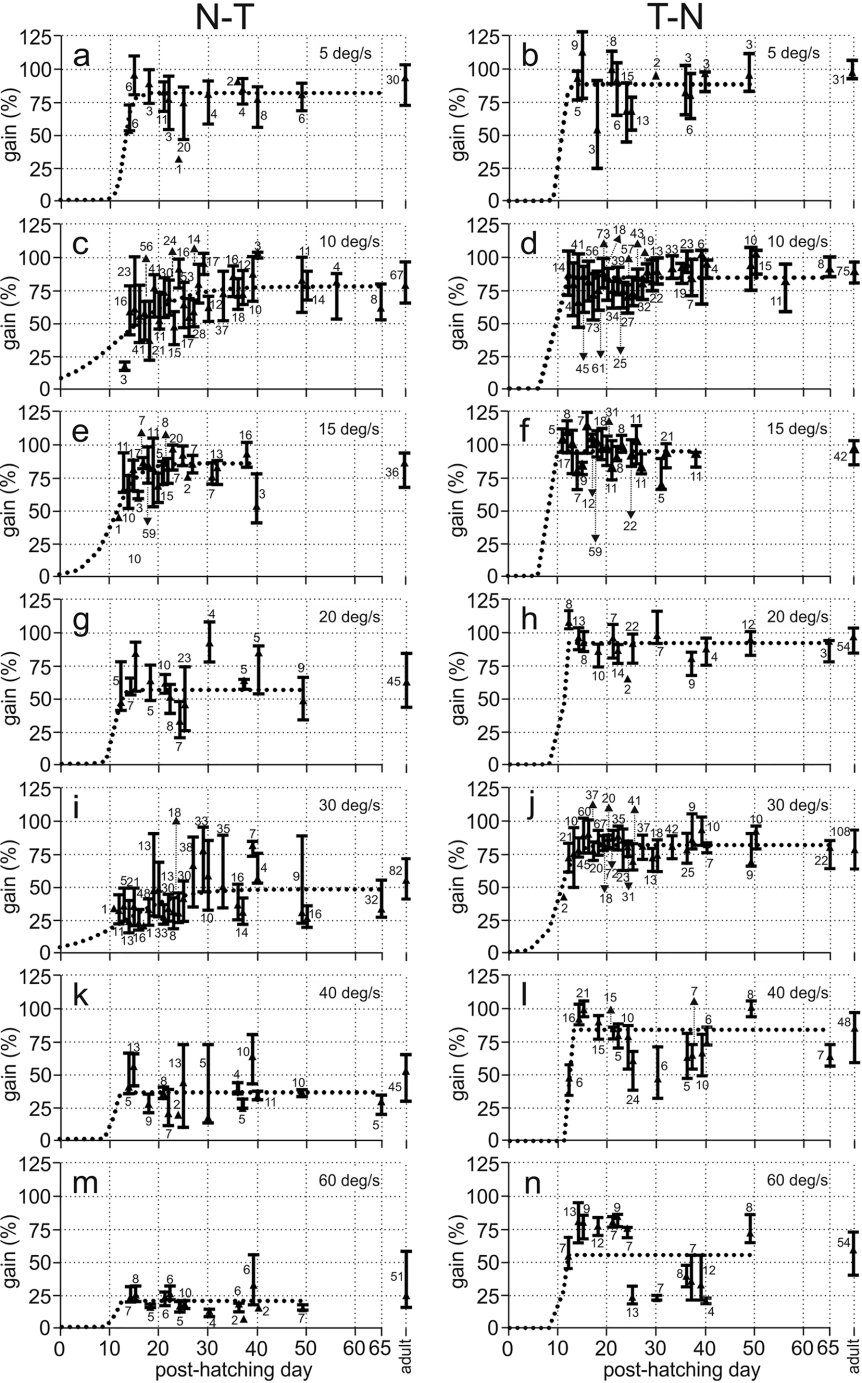
Dependence of monocular gains on age. **a**–**n** All monocular data shown as recorded for different velocities (row) and either N–T (left column) or T–N (right column) stimulation together with the fit functions (dotted lines). Specifications are as explained in the legend to Fig. 4. Note the lower gains and the delayed development for N–T responses compared with T–N responses

**Fig. 7:**
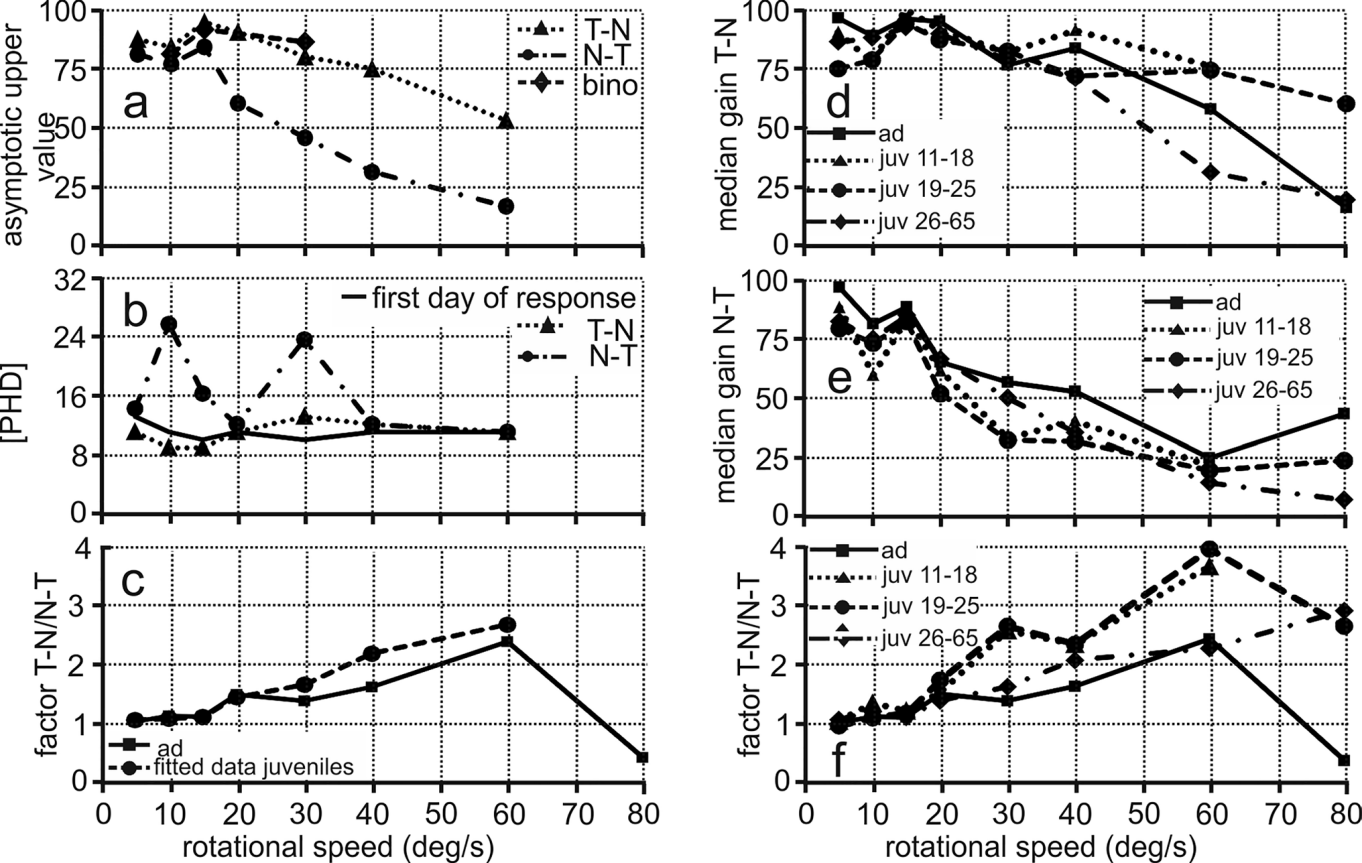
Quantification of asymmetry. **a** Fit parameter “asymptotic upper value”, **b** First day of response and 90%-PHDs. The “first day of response” (for a definition see text) refers to both T–N and N–T conditions and is documented for each stimulus velocity. The 90%-PHDs are separately plotted for T–N and N–T stimulation. **d**, **e** Data from three developmental periods (P11–P18, P19–P25, P26–P65). **c**, **f** Asymmetry factors T–N/N–T in juveniles (juv) derived from the fits (**c**) and from the data (**f**) in comparison to the measured adult factors (ad)

The quantitative analysis of the data sets for stimulus velocities of 10, 15, and 30 deg/s (Fig. 4) demonstrated that adult-like gain values were reached very early. The median gains reached an adult-like value from the first day of response. For example, the first day of response for 30 deg/s was on P11 in owl K (Fig. 4f). Already at this age, the gain was not statistically different from the gain at P33 (Mann–Whitney *U* test, number of cases P11: 6, P33: 11 (*U* = 32, *z* score = 0.05025, *p* = 0.96012). Non-significant differences were also observed for the first and last days for which we have data in the other two owls (Mann–Whitney *U* test, number of cases owl F: P19: 17, P26: 14 (*U* = 99, *z* score: 0.7647, *p* = 0.4444); owl J: Mann–Whitney *U* test, number of cases P14: 23, P40: 8 (*U* = 49.5, *z* score =  − 1.8967, *p* = 0.05787)). Data from all owls (owls F, K, J) were similar (Fig. 4e). The data recorded during the whole juvenile period were pooled and tested against the data from adult birds as published in Wagner et al. (2021). There was no difference between the two data sets (Mann–Whitney *U* test, number juveniles: 407, number adults: 73, *U* = 15,073, *z* score = 0.1989, *p* = 0.8424; see also Fig. 5). The time course of development was fitted by a sigmoidal function (which was chosen as it describes also the monocular data (Figs. 6, 7), see Material and methods). The function fitting the 30 deg/s data demonstrated that the 90%-PHD corresponded to the first day of response (Fig. 4e, f).

Similar observations were made for a velocity of 15 deg/s for which data from owls G and H were available. The earliest recording in owl H, at P13, already yielded data (median gain value 87.8) that was statistically not different from the data at P32 (median gain value 91.1) (Mann–Whitney *U* test, number of cases P13: 15, P32: 20, *U* = 141, *z* score = 0.28333, *p* = 0.77948). These observations were supported when the data of owls G and H were pooled (Fig. 4c). Again, juvenile and adult data were not different (Mann–Whitney *U* test, number juveniles: 211, number adults: 11, *U* = 1046.5, *z* score = − 0.5224, *p* = 0.6015; see also Fig. 5).

The data for a stimulus velocity of 10 deg/s were mainly based on recordings with owl I (Fig. 4b). Some data came also from owls F, J, and K (Fig. 4a). Again, the very first recordings, on P14, showed a median gain (84.3) close to that measured at P29 (85.4) or P 33 (92.2). These median gains were much higher than that determined at P56 (72). For 10 deg/s stimulus velocity, the juvenile data yielded significantly lower gains than measured in adults (Mann–Whitney *U* Test, number juveniles: 634, number adults: 64, *U* = 9867, *z* score = − 6.7781, *p* = 1.218*10^– 11^; see also Fig. 5). The reason for this difference is not clear. For all other stimulus velocities tested, the juvenile and the adult responses were not different (Fig. 5).

Median gains with binocular stimulation were close to 100% for velocities up to 20 deg/s (Fig. 5). The median gains decreased to 70% for velocities up to 60 deg/s and to about 40% for a stimulus velocity of 80 deg/s (Fig. 5). Gain values did not change during development. There were some extraordinary recording days, with median values either below (Fig. 4a, b, P17 and P18) or above (Fig. 4e, P36) the rest of the values. The differences between the 1st and the 3rd quartiles were between 14.3 and 24.2 percent of gain in absolute terms. The differences amounted to 15–29%, determined relative to the median gain values. In summary, binocular gains measured in juvenile birds were not statistically different from adult gains for 6 out of 7 stimulus velocities tested that ranged from 5 to 60 deg/s (Fig. 5).

### Monocular optocollic responses of juvenile barn owls

The response pattern for monocular stimulation was more complex than the responses to binocular stimulation. Major differences occurred in the responses to N–T and T–N stimulation. The first PHD at which the birds responded was P11 (in owl K, stimulus velocity: 30 deg/s). The responses to both T–N and N–T stimulation were short and of low gain at this PHD (Fig. 3b, c; see Fig. 6i, j for a quantitative analysis of the reaction with a stimulus velocity of 30 deg/s). This changed fast for the responses to T–N stimulation, both for 10 deg/s (Fig. 3n, Fig. 6d), and for 30 deg/s (Fig. 3e, h, k; Fig. 6j). By contrast, gains to N–T stimulation remained low for several days. These gains gradually increased during development. At P19 responses to N–T stimulation were of high gain for a stimulus velocity of 10 deg/s (Fig. 3o: single gain values 81.5 and 85, quantitative analysis in Fig. 6c: median gain: 76.8). At this PHD, gains were still low for 30 deg/s (Fig. 3i: single gain values: 26 and 31, quantitative analysis in Fig. 6i: median gain: 47). At P27, gains for N–T stimulation had increased also for a stimulus velocity of 30 deg/s (Fig. 3l: single gain values: 71, 56, 71, 70.7, 64.5, quantitative analysis in Fig. 6i: median gain: 66).

The fitting of the responses provided insights into the duration of development. The inflection points of the fit function (parameter *b* in Eq. 2) were all between P9 and P13, which suggested that the development began at similar times for all velocities and conditions. The duration of development may be derived from the 90–50 differences and the 90%-PHDs. These two parameters are related to the factor *c* of the fitting function. They varied a lot with stimulus velocity (e.g., range 10–26 days for 90%PHD, Fig. 7b). They yielded highly correlated values over the 7 stimulus velocities used (7 data points, correlation coefficient: 0.988, *p* < 0.00003). In the following, we arbitrarily use the 90%-PHDs as a measure for the duration of the development (Fig. 7b). The 90%-PHDs for N–T stimulation were 26, 17, and 24 for stimulus velocities of 10, 15, and 30 deg/s, respectively (Fig. 7b). The responses to T–N stimulation were high from very early on. The 90%-PHDs for T–N stimulation were between P11 and P14 for all stimulus velocities tested (Fig. 7b). In other words, the 90%-PHD was reached almost immediately after the first day of response (Fig. 7b). The longest time necessary to reach 90% of the final values with T–N stimulation was three days. This occurred for a stimulus velocity of 30 deg/s (Fig. 7b).

The fitting of the data not only made it possible to quantify the duration of development, but also provided insight into the differences in upper gain values for the different stimulus types (binocular, monocular T–N, monocular N–T). For binocular stimulation, sufficient data for fitting were available for 10, 15 and 30 deg/s. The comparisons showed that the upper values for binocular stimulation were very close to the upper values for T–N stimulation (compare dashed and dotted lines in Fig. 7a). Larger differences were seen between the responses to binocular and T–N stimulation on the one and the responses to N–T stimulation on the other side (Fig. 7a). The upper values for N–T responses were significantly lower than the upper values for T–N responses (7 pairs of upper values, Wilcoxon Matched Pairs signed rank test, z = − 2.418; *p* = 0.016).

Figure 7a shows an increase of the differences in the upper values for T–N and N–T responses with stimulus velocity. This resulted in an increase of the T–N/N–T factors with stimulus velocity (Fig. 7c). The juvenile T–N/N–T factors derived from the fits are very similar to the measured T–N/N–T factors in adults for velocities up to 20 deg/s. The earlier factors are slightly higher than the latter factors for higher velocities.

The upper values of the fits yielded data that reflected the final result of development. Additionally, it was also interesting to examine the temporal change of the gains and specifically the T–N/N–T factors during development. To this end, we pooled data from three distinct age periods (P11–P18, P19–P25, and P26–P65). We compared the results from the juveniles with those from the adults (Fig. 7d–f). We are aware that the pooling coarsened the time resolution of the data compared to the data shown in Fig. 6. However, the resulting curves are smoother, and allow better insight into the underlying mechanisms than the plots shown in Fig. 6. Figure 7d demonstrates that the T–N gains were high from early on. T–N gains for a stimulus velocity of 60 and 80 deg/s decreased in the last period (Fig. 7d). Note, however, that the latter data points are based on low numbers (Table 3). Gains for N–T stimulus did not change much for stimulus velocities up to 20 deg/s and also not for 40 and 60 deg/s (Fig. 7e) in the course of development. The gain for a stimulus velocity of 30 deg/s increased in the last period ranging from P26-P65 compared to the gains in the earlier two periods and reached an adult-like value (Fig. 7e). Figure 7f summarizes the data shown in Fig. 7d and e. This plot demonstrates that the measured factors T–N/N–T for velocities up to 20 deg/s were close to 1 and adult-like from the first period on. By contrast, there were developmental changes of the factors T–N/N–T for velocities above 20 deg/s. The values were larger than the adult values for the first two time-averaging periods from P11 to P18 and P19 to P25. The T–N/N–T factors derived from the measured gain data reached adult-like values for the last analysis period (P26–P65) (Fig. 7f). This observation is consistent with the T–N/N–T factors derived from the fitted data shown in Fig. 7c.

## Discussion

### Methodology and behavioral variability

We have already discussed methodology in the work on the adult barn owls (Wagner et al. 2021), and the considerations detailed in the earlier study hold also for this study.

Similar to what was reported by Simon (1954), owls up to about P13 supported their head on the rim of their container. This did not appear to influence the reaction of the birds to the stimulus. Binocular gains were adult-like from P11 on. In contrast to Simon (1954) we did not see a leaning of the head to one side, if one eye was occluded. This may be a difference between a bird with lateral eyes like the chicken, and the barn owl which has frontal eyes.

A potential weakness in our study is that the data vary considerably between the different stimulus velocities. To address this, we based the conclusions on data obtained with stimulus velocities of 10 deg/s, 15 deg/s, and 30 deg/s. The data recorded with the other stimulus velocities supplemented these observations, and we note that the development of responses to stimulus velocities above 30 deg/s remains an open question.

Although the optomotor reflex very reliably elicits a following behavior, the responses are variable. For example, in pigeons the responses differ whether the head is fixed or free (Gioanni 1988), or whether the animal is in the resting, standing, walking or flying condition (Maurice et al. 2006). Since we used untrained birds, the variability we observed in the responses of the juveniles was not surprising. Variability may be reduced in experiments with trained owls (van der Willigen et al. 1998; Nelson and Takahashi 2010; Kettler et al. 2017; Zahar et al. 2018). In such settings, it is possible to test whether a subject is under stimulus control (Green and Swets 1966). This was not possible in our experiments with the juvenile owls in which we obtained data just after opening of the eyes.

The variability within one daily recording was typically around 25% of gain. For low stimulus velocities with gains close to 100%, a variability of 25% corresponds to a coefficient of variation of 0.25. This value is similar to the variability observed in sound-localization tasks (Wagner 1993; Hausmann et al. 2009). A coarse reconstruction of the data presented by Wallman and Velez (1985, their Fig. 3), Distler et al. (1999, their Fig. 2), and Maurice et al. (2006, their Fig. 4) showed a similar variability in juvenile chickens, macaques and adult pigeons in tests of optomotor responses.

### Development of optocollic responses in barn owls compared with other species

The ontogenetic change in optocollic gains of owls was rapid, although responses showed different rates of development with respect to stimulus velocity, stimulus direction, and stimulus type. This is similar to the changes observed in primates (Roy et al. 1989; Distler et al. 1999; Distler and Hoffmann 2011) and cats (Distler and Hoffmann 1992, 2003). The developmental time course in the owl was also similar to that observed in non-human primates (Distler et al. 1999; Distler and Hoffmann 2011). Adult-like binocular responses were seen in juvenile owls from the first day of response. Monocular responses to stimulation in the T–N direction attained adult-like values also within a few days after the eyes opened. By contrast, monocular responses to N–T stimulation took a few days longer to reach adult-like values.

Symmetry of the monocular hOCR was reached for a stimulus velocity of 30 deg/s by a stable high gain in the T–N direction and a temporally increasing gain in the N–T direction. These findings are again similar to what was observed in mammals with frontal eyes (Naegele and Held 1982; Distler and Hoffmann 1992, 2003; Distler et al. 1999). By contrast, the adult-like T–N/N–T factors for stimulus velocities above 30 deg/s were mainly due to decreased gains in the T–N direction. This is contrary to what was expected from the above-cited studies in cats and primates. However, the data for 40, 60, and 80 deg/s are less reliable. Therefore, more data are necessary to find out whether this constitutes a second way to reach symmetry.

The only studies on the optomotor responses in young birds we found were those of Simon (1954) and of Wallman and Velez (1985). Both Simon (1954) and Wallman and Velez (1985) observed an increase of the asymmetry of the monocular responses with time. The latter authors argued that an increased asymmetry in the older chickens may be related to the lateral position of the eyes in this bird. They also speculated that asymmetry may be the more functional state in lateral-eyed animals. We observed much more symmetric monocular responses in both juvenile (this study) and adult (Wagner et al. 2021) owls than Wallman and Velez (1985) observed in chickens. In the owls, the asymmetry was weaker for lower than for higher stimulus velocities. This is similar to what Wallman and Velez (1985) reported from chickens.

### The neural circuits underlying optocollic, optokinetic, and optomotor responses

The visually induced wide-field responses are driven by a sub-cortical network (Grasse et al. 1984; Schor 1993; Wallman 1993; Distler et al. 2002). The network receives direct input from the retina, sub-cortical inputs via the optic tectum, and indirect inputs from the cortex or its avian analog, the visual Wulst (for reviews see Wallman 1993; Wylie et al. 2014). Important nuclei in mammals are the nucleus of the optic tract and the terminal nuclei (Schor 1993; Masseck and Hoffmann 2009). The homologous nuclei in birds are the nucleus lentiformis mesencephali and the nucleus of the basal optic root (nBOR) (Rio et al. 1983; Wylie et al. 2005). The OKR in response to high velocities is mainly controlled by cortical input in both N–T and T–N directions. By contrast, OKR to low velocities is mediated in the N–T direction by the cortex and in the T–N direction by both the cortex and sub-cortical pathways (Montarolo et al. 1981; Grasse et al. 1984). In young kittens, the nucleus of the optic tract only receives input from the contralateral retina; these inputs drive the responses with T–N stimulation. In older kittens, this nucleus receives also a cortical input. This input makes functional synapses in the nucleus at the time when symmetry of the OKR is first seen (Distler and Hoffmann, 1992). In the chicken, directional sub-regions in nBOR are not present at hatching but develop rapidly within the first postnatal weeks (McKenna and Wallman 1985). We speculate that a similar development as in the chicken nBOR occurs also in juvenile owls. It would also be interesting to determine whether response properties of optomotor neurons in barn owls resemble those in frontal-eyed mammals (Distler and Hoffmann 2011), to what Wylie et al. (1994) demonstrated for saw-whet owls, to lateral-eyed birds (Morgan and Frost 1981; Crowder et al. 2003), to specialists like hummingbirds (Gaede et al. 2016), or have established their own specific distribution.

## Supplementary Information

Below is the link to the electronic supplementary material.

Supplementary file1 (AVI 2148 KB)

Supplementary file2 (AVI 2488 KB)

Supplementary file3 (AVI 2710 KB)

Supplementary file4 (AVI 8573 KB)

## Abbreviations

P or PHD: Post-hatching day
90%-PHD: PHD at which 90% of the upper (asymptotic) value of the fit function is reached
90–50-difference: Difference in days between the 90%-PHD and the PHD of the inflection point of the fit function (50%)
deg: Degrees
hOCR: Horizontal optocollic response
nBOR: Nucleus of the basal optic root
N–T: Nasal-to-temporal
OCR: Optocollic response
OKR: Optokinetic response
OMR: Optomotor response
RMSE: Root-mean square error
T–N: Temporal-to-nasal

## References

[CR1] Bunn DS, Warburton AB, Wilson RDS (1982). The barn owl.

[CR2] Carde RT (2021). Navigation along windborne plumes of pheromone and resource-linked odors. Ann Rev Entomol.

[CR3] Carpenter RHS (1988). Movements of the eyes.

[CR4] Crowder NA, Dawson MR, Wylie DR (2003). Temporal frequency and velocity-like tuning in the pigeon accessory optic system. J Neurophysiol.

[CR5] Distler C, Hoffmann KP (1992). Early development of the subcortical and cortical pathway involved in optokinetic nystagmus: the cat as a model for man?. Beh Brain Res.

[CR6] Distler C, Hoffmann KP (2003). Development of the optokinetic response in macaques: a comparison with cat and man. Ann N Y Acad Sci.

[CR7] Distler C, Hoffmann KP (2011). Visual pathway for the optokinetic reflex in infant macaque monkeys. J Neurosci.

[CR8] Distler C, Mustari MJ, Hoffmann KP (2002). Cortical projections to the nucleus of the optic tract and dorsal terminal nucleus and to the dorsolateral pontine nucleus in macaques: a dual retrograde tracing study. J Comp Neurol.

[CR9] Distler C, Vital-Durand F, Korte R, Korbmacher H, Hoffmann KP (1999). Development of the optokinetic system in macaque monkeys. Vision Res.

[CR10] Du Lac S, Knudsen EI (1990). Neural maps of head movement vector and speed in the optic tectum of the barn owl. J Neurophysiol.

[CR11] Gaede AH, Goller B, Lam JPM, Wylie DR, Altshuler DL (2016). Neurons responsive to global visual motion have unique tuning properties in hummingbirds. Curr Biol.

[CR12] Gioanni H (1988). Stabilizing gaze reflexes in the pigeon (*Columba livia*): I. Horizontal and vertical optokinetic eye (OKN) and head (OCR) reflexes. Exp Brain Res.

[CR13] Gioanni H, Rey J, Villalobos J, Bouyer JJ, Gioanni Y (1981). Optokinetic nystagmus in the pigeon (*Columba livia*). I. Study in monocular and binocular vision. Exp Brain Res.

[CR14] Grasse KL, Cynader MS, Douglas RM (1984). Alterations in response properties in the lateral and dorsal terminal nuclei of the cat accessory optic system following visual cortex lesions. Exp Brain Res.

[CR15] Green DM, Swets JA (1966). Signal detection theory and psychophysics.

[CR16] Hausmann L, von Campenhausen M, Endler F, Singheiser M, Wagner H (2009). Improvements of sound-localization capabilities by the facial ruff of the barn owl (*Tyto alba*) as demonstrated by virtual ruff removal. PLoS ONE.

[CR17] Huang YY, Neuhauss S (2008). The optokinetic response in zebrafish and its applications. Front Biosci.

[CR18] Iwaniuk AN, Heesy CP, Hall MI, Wiley DR (2008). Relative Wulst volume is correlated with orbit orientation and binocular visual field in birds. J Comp Physiol A.

[CR19] Kettler L, Griebel H, Ferger R, Wagner H (2017). Combination of interaural level and time difference in azimuthal sound localization in owls. Eneuro.

[CR20] Knapp CM, Proudlock FA, Gottlob I (2013). OKN asymmetry in human subjects: a literature review. Strabismus.

[CR21] Koeppl C, Futterer E, Nieder B, Sistermann R, Wagner H (2005). Embryonic and posthatching development of the barn owl (*Tyto alba*): reference data for age determination. Dev Dyn.

[CR22] Krings M, Rosskamp L, Wagner H (2018). Development of ear asymmetry in the American barn owl (*Tyto furcata pratincola*). Zoology.

[CR23] Lewis TL, Maurer D, Chung JY, Holmes-Shannon R, van Schaik CS (2000). The development of symmetrical OKN in infants: quantification based on OKN acuity for nasalward versus temporalward motion. Vision Res.

[CR24] Masseck OA, Hoffmann KP (2009). Comparative neurobiology of the optokinetic reflex. Ann N Y Acad Sci.

[CR25] Maurice M, Gioanni H, Abourachid A (2006). Influence of the behavioural context on the optocollic reflex (OCR) in pigeons (*Columba livia*). J Exp Biol.

[CR26] McKenna OC, Wallman J (1985). Functional postnatal changes in avian brain regions responsive to retinal slip: A 2-deoxy-d-glucose study. J Neurosci.

[CR27] Montarolo PG, Precht W, Strata P (1981). Functional organization of the mechanisms subserving the optokinetic nystagmus in the cat. Neuroscience.

[CR28] Morgan B, Frost BJ (1981). Visual response characteristics of neurons in nucleus of the basal optic root of pigeons. Exp Brain Res.

[CR29] Mowrer OH (1936). A comparison of the reaction mechanisms mediating optokinetic nystagmus in human beings and in pigeons. Psychol Monogr.

[CR30] Naegele JR, Held R (1982). The postnatal development of monocular optokinetic nystagmus in infants. Vision Res.

[CR31] Nalbach HO (1992). Translational head movements of pigeons in response to a rotating pattern: characteristics and tool to analyse mechanisms underlying detection of rotational and translational optic flow. Exp Brain Res.

[CR32] Nelson BS, Takahashi TT (2010). Spatial hearing in echoic environments: the role of the envelope in owls. Neuron.

[CR33] Netser S, Ohayon S, Gutfreund Y (2010). Multiple manifestations of microstimulation in the optic tectum: eye movements, pupil dilations, and sensory priming. J Neurophysiol.

[CR34] Nieder A, Wagner H (2000). Horizontal-disparity tuning of neurons in the visual forebrain of the behaving barn owl. J Neurophysiol.

[CR35] Rio JP, Villalobs J, Miceli D, Reperant J (1983). Efferent projection of the visual Wulst upon the nucleus of the basal optic root in the pigeon. Brain Res.

[CR36] Roulin A (2020). Barn owls Evolution and Ecology.

[CR37] Roy MS, Lachapelle P, Lepore F (1989). Maturation of the optokinetic nystagmus as a function of the speed of stimulation in full term and preterm infants. Clin vis Sci.

[CR38] Schor CM (1993). Development of OKN. Rev Oculomot Res.

[CR39] Shawyer C (1998). The barn owl.

[CR40] Simon ME (1954). Der optomotorische Nystagmus während der Entwicklung normaler und optisch isoliert aufwachsender Küken. Z Vergl Physiol.

[CR41] Steinbach MJ, Money KE (1973). Eye movements of the owl. Vision Res.

[CR42] Türke W, Nalbach HO, Kirschfeld K (1996). Visually elicited head rotation in pigeons. Vision Res.

[CR43] Van den Berg AV, Collewijn H (1988). Directional asymmetries of human optokinetic nystagmus. Exp Brain Res.

[CR44] Van der Willigen R, Frost BJ, Wagner H (1998). Stereoscopic depth perception in the owl. NeuroReport.

[CR45] Wagner H (1993). Sound localization deficits induced by lesions in the barn owl’s auditory space map. J Neurosci.

[CR46] Wagner H, Pappe I, Nalbach HO (2021). Optocollic responses in adult barn owls (*Tyto furcata*). J Comp Physiol A.

[CR47] Wallman J, Miles FA, Wallman J (1993). Subcortical optokinetic mechanisms. Visual motion and its role in the stabilization of gaze.

[CR48] Wallman J, Velez J (1985). Directional asymmetries of optokinetic nystagmus: developmental changes and relation to the accessory optic system and to the vestibular system. J Neurosci.

[CR49] Wylie DR, Shaver SW, Frost BJ (1994). The visual response properties of neurons in the nucleus of the basal optic root of the northern Saw-whet owl (*Aegolius acadicus*). Brain Behav Evol.

[CR50] Wylie DR, Ogilvie CJ, Crowder NA, Barkley RR, Winship IR (2005). Telencephalic projections to the nucleus of the basal optic root and pretectal nucleus lentiformis mesencephali in pigeons. Vis Neurosci.

[CR51] Wylie DR, Kolominsky J, Graham DJ, Lisney TJ, Gutierrez-Ibanez C (2014). Retinal projection to the pretectal nucleus lentiformis mesencephali in pigeons (*Columba livia*). J Comp Neurol.

[CR52] Zahar Y, Levi-Ari T, Wagner H, Gutfreund Y (2018). Behavioral evidence and neural correlates of perceptual grouping by motion in the barn owl. J Neurosci.

